# Catabolism of 2-keto-3-deoxy-galactonate and the production of its enantiomers

**DOI:** 10.1007/s00253-024-13235-x

**Published:** 2024-07-02

**Authors:** Eun Ju Yun, Sun-Hee Lee, Subin Kim, Hae Seul Ryu, Kyoung Heon Kim

**Affiliations:** 1 Division of Biotechnology, Jeonbuk National University, Iksan, 54596 Republic of Korea; 2 Department of Biotechnology, Graduate School, Korea University, Seoul, 02841 Republic of Korea

**Keywords:** 2-Keto-3-deoxy-galactonate, Catabolism, Stereospecificity, Enantiomer, Pyruvate, Glyceraldehyde

## Abstract

**Abstract:**

2-Keto-3-deoxy-galactonate (KDGal) serves as a pivotal metabolic intermediate within both the fungal d-galacturonate pathway, which is integral to pectin catabolism, and the bacterial DeLey-Doudoroff pathway for d-galactose catabolism. The presence of KDGal enantiomers, l-KDGal and d-KDGal, varies across these pathways. Fungal pathways generate l-KDGal through the reduction and dehydration of d-galacturonate, whereas bacterial pathways produce d-KDGal through the oxidation and dehydration of d-galactose. Two distinct catabolic routes further metabolize KDGal: a nonphosphorolytic pathway that employs aldolase and a phosphorolytic pathway involving kinase and aldolase. Recent findings have revealed that l-KDGal, identified in the bacterial catabolism of 3,6-anhydro-l-galactose, a major component of red seaweeds, is also catabolized by *Escherichia coli*, which is traditionally known to be catabolized by specific fungal species, such as *Trichoderma reesei*. Furthermore, the potential industrial applications of KDGal and its derivatives, such as pyruvate and d- and l-glyceraldehyde, are underscored by their significant biological functions. This review comprehensively outlines the catabolism of l-KDGal and d-KDGal across different biological systems, highlights stereospecific methods for discriminating between enantiomers, and explores industrial application prospects for producing KDGal enantiomers.

**Key points:**

*• KDGal is a metabolic intermediate in fungal and bacterial pathways*

*• Stereospecific enzymes can be used to identify the enantiomeric nature of KDGal*

*• KDGal can be used to induce pectin catabolism or produce functional materials*

## Introduction

Diverse ecological niches, ranging from terrestrial to marine environments, harbor distinct resources. For instance, pectin is commonly present in fruits such as citrus, apples, and grapes, while agar is predominantly sourced from red seaweeds (*Rhodophyta*). The catabolic pathways responsible for degrading the monomeric constituents of these polysaccharides, such as d-galacturonate, a primary component of pectin, as well as d-galactose and 3,6-anhydro-l-galactose (AHG), key constituents of red seaweeds, have been elucidated in filamentous fungi and marine bacteria, respectively (Peltonen and Richard 2022; Tästensen et al. 2020; Yun et al. 2023).

2-Keto-3-deoxy-galactonate (KDGal), which exists in both l-KDGal and d-KDGal enantiomeric forms, serves as a crucial metabolic intermediate in the degradation of d-galacturonate, d-galactose, and AHG (Peltonen and Richard 2022; Tästensen et al. 2020; Yun et al. 2023). l-KDGal is generated through distinct pathways in fungi and bacteria. In fungi, the catabolism of d-galacturonate to l-KDGal involves a sequence of reduction and dehydration reactions, first discovered in the filamentous fungi *Trichoderma reesei* and *Aspergillus niger* (Peltonen and Richard 2022; Wiebe et al. 2010). In bacteria, l-KDGal is produced from AHG through dehydrogenation and cycloisomerization processes, initially identified in the marine bacterium *Vibrio* sp. EJY3 (Yun et al. 2015, 2023). The formation of d-KDGal entails the oxidation and subsequent dehydration of d-galactose, a reaction observed in bacterial and archaeal oxidative d-galactose pathways (Deacon and Cooper 1977; Tästensen et al. 2020; Wong and Yao 1994).

The catabolism of l-KDGal and d-KDGal varies among species, notably between fungi and bacteria, demonstrating the diversity of metabolic pathways. In fungi such as *T. reesei* and *A. niger*, l-KDGal undergoes a distinct nonphosphorolytic breakdown (Hilditch et al. 2007; Li et al. 2022). This pathway cleaves l-KDGal into l-glyceraldehyde and pyruvate (Hilditch et al. 2007; Li et al. 2022). Pyruvate plays a crucial role in cellular metabolism as it can directly feed into the tricarboxylic acid (TCA) cycle, facilitating the production of energy molecules. Concurrently, l-glyceraldehyde is converted into glycerol, demonstrating the versatility of fungal metabolic processes in utilizing l-KDGal for energy generation and cellular functions (Li et al. 2022).

The metabolic fate of d-KDGal in bacteria, as exemplified by *Escherichia coli* and *Azotobacter vinelandii*, follows a phosphorolytic catabolic pathway (Deacon and Cooper 1977; Peabody et al. 2019; Wong and Yao 1994). Within this pathway, d-KDGal undergoes transformation into d-KDGal 6-phosphate, which subsequently splits into d-glyceraldehyde 3-phosphate and pyruvate. This process not only exemplifies the biochemical flexibility of bacteria in metabolizing sugars but also connects d-KDGal catabolism to central carbon metabolism, given that both resulting metabolites are integral components of glycolysis (Deacon and Cooper 1977; Peabody et al. 2019; Wong and Yao 1994). Archaea exhibit a more versatile approach to d-KDGal catabolism, engaging both phosphorolytic and nonphosphorolytic pathways. This dual-pathway strategy, documented in recent research (Kopp et al. 2020; Tästensen et al. 2020), underscores the metabolic diversity across microbial life forms and suggests a broader range of adaptability to energy and carbon utilization.

KDGal plays a critical but often overlooked role in the catabolism of terrestrial and marine bioresources, such as pectin and red seaweeds, as identified in recent studies (Roman-Benn et al. 2023; Yun et al. 2023). The transient nature of KDGal as a metabolic intermediate may have led to its catabolic pathways being less explored in scientific literature. Moreover, the industrial potential of KDGal, including its enantiomers l-KDGal and d-KDGal and their derived products, remains underexplored and undervalued.

This review aimed to fill these gaps by providing an exhaustive analysis of the catabolic pathways of l-KDGal and d-KDGal in various biological systems. It delves into the specific enzyme functions critical to these pathways and offers a detailed examination of the stereospecific methods used to distinguish the enantiomeric forms of these compounds. In addition, this review focuses on the untapped industrial potential of KDGal and its derivatives, emphasizing the significance of their enantiomeric structures in bioactivity and pharmacological efficacy. By elucidating the distinct bioactivities of each enantiomeric form of KDGal and its derivatives, this review highlights their potential applications in diverse industrial sectors.

## Catabolism of l-KDGal

### Fungal catabolic pathway of d-galacturonate

**Fig. 1:**
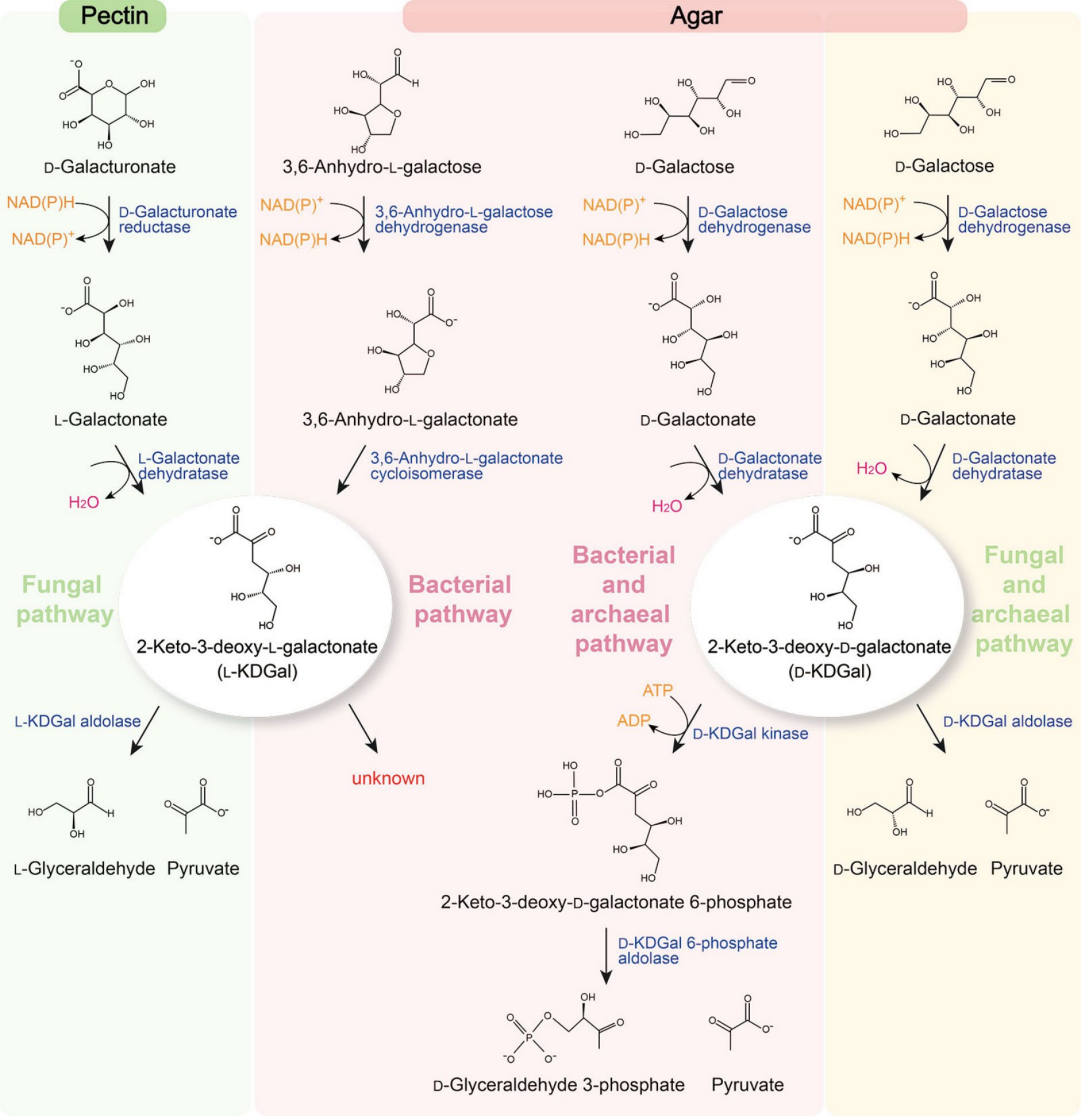
l-KDGal and d-KDGal existing as the metabolic intermediates in the catabolic pathways in d-galacturonate, 3,6-anhydro-l-galactose, and d-galactose in certain species belonging to fungi, bacteria, and archaea. l-KDGal is found in the fungal d-galacturonate and bacterial 3,6-anhydro-l-galactose pathways. Moreover, d-KDGal is found in the bacterial, fungal, and archaeal catabolic pathway of d-galactose. l-KDGal and d-KDGal are then further catabolized via nonphosphorolytic or phosphorolytic pathways

l-KDGal’s role as a metabolic intermediate in the fungal degradation of d-galacturonate, the most prevalent hexuronic acid and a primary constituent of pectin, was initially identified in key studies by Kuorelahti et al. (2005, 2006) and Wiebe et al. (2010). In fungi such as *T. reesei* and *A. niger*, d-galacturonate is first converted into l-galactonate through the action of NAD(P)H-dependent d-galacturonate reductase (Kuorelahti et al. 2005; Wiebe et al. 2010). This is followed by the conversion of l-galactonate to l-KDGal by l-galactonate dehydratase (Kuorelahti et al. 2006; Wiebe et al. 2010) (Fig. 1). Subsequently, l-KDGal is catabolized into pyruvate and l-glyceraldehyde by l-KDGal aldolase, a process further elaborated by Alazi et al. (2017) and Hilditch et al. (2007) (Fig. 1). Pyruvate seamlessly integrates into central carbon metabolism, whereas l-glyceraldehyde is converted to glycerol by l-glyceraldehyde reductase (Alazi et al. 2017; Hilditch et al. 2007) (Fig. 1).

In the initial phase of the d-galacturonate pathway, d-galacturonate reductases from *T. reesei* and *A. niger* display a marked preference for NADPH over NADH, highlighting an enzymatic bias toward specific cofactors (Kuorelahti et al. 2005; Wiebe et al. 2010). However, recent research by Peltonen and Richard (2022) revealed that the *gaa1* gene, which encodes a d-galacturonate reductase from *Euglena gracilis*, exhibits a similar affinity for both NADPH and NADH. This discovery suggests that *gaa1* from *E. gracilis* could be harnessed on engineered microbial platforms to efficiently enhance the utilization of pectin-rich biomass.

### Bacterial catabolic pathway of 3,6-anhydro-l-galactose

Previously, l-KDGal was considered exclusive to fungal catabolic pathways, with no known bacterial mechanisms involved in its production. This notion was reversed by discovering a catabolic pathway for AHG in *Vibrio* sp. EJY3, revealing a bacterial route to l-KDGal (Yun et al. 2015, 2023). AHG, a predominant sugar in red seaweeds alongside d-galactose (Araki 1956; Yun et al. 2015), undergoes a two-step conversion to l-KDGal. This process begins with the oxidation to 3,6-anhydro-l-galactonate by NAD(P)^+^-dependent AHG dehydrogenase, followed by its conversion to KDGal by 3,6-anhydro-l-galactonate cycloisomerase (Yu et al. 2022; Yun et al. 2015) (Fig. 1).

Recent findings have confirmed that KDGal, produced through the AHG catabolic pathway, is present in its l-form (Yun et al. 2023) (Fig. 1). Intriguingly, *E. coli*, a widely used industrial microbe, can utilize l-KDGal (Yun et al. 2023). Compounds such as l-KDGal and 2-keto-3-deoxy-gluconate (KDGlc), which appear solely as metabolic intermediates in both fungal and bacterial pathways, cannot initiate their transport systems because external 2-keto-3-deoxy-hexonate is unable to induce these systems (Lagarde et al. 1973; Pouyssegur and Stoeber 1974). However, when l-KDGal is introduced alongside d-galacturonate, which serves as an inducer of the transport system, *E. coli* can utilize l-KDGal, as evidenced by increased cell growth in the presence of l-KDGal compared with d-galacturonate alone. This was confirmed by monitoring l-KDGal concentrations before and after *E. coli* cultivation (Yun et al. 2023).

Although the specificity of how l-KDGal integrates into *E. coli*’s central carbon metabolism remains to be elucidated (Fig. 1), it is hypothesized that l-KDGal may be converted into d-KDGal or d-KDGlc. These compounds would then undergo catabolism via a phosphorolytic pathway involving 2-keto-3-deoxy-hexonate kinase and 2-keto-3-deoxy-hexonate 6-phosphate aldolase to yield d-glyceraldehyde 3-phosphate and pyruvate (Deacon and Cooper 1977; Martis et al. 2021). Further studies are required to fully understand bacterial l-KDGal catabolism and its implications.

## Catabolism of d-KDGal

### Bacterial oxidative d-galactose pathway

The Leloir pathway is the predominant mechanism of d-galactose catabolism and features three key enzymatic reactions: galactokinase, galactose 1-phosphate uridylyltransferase, and phosphoglucomutase. These enzymes collectively convert galactose into glucose 6-phosphate, an intermediary metabolite integral to the Embden-Meyerhof-Parnas and pentose phosphate pathways (Holden et al. 2003; Tästensen et al. 2020).

d-KDGal has been identified as an intermediate in the oxidative galactose pathway, also known as the DeLey-Doudoroff (DD) pathway. This alternative route is utilized by bacteria, including *E. coli* and *A. vinelandii*, for the catabolism of d-galactose (Deacon and Cooper 1977; Wieczorek et al. 1999; Wong and Yao 1994). Initially, d-galactose is oxidized to d-galactonate by NAD(P)^+^-dependent d-galactose dehydrogenase, followed by its conversion to d-KDGal via d-galactonate dehydratase (Fig. 1). Additionally, enzymes from *Burkholderia ambifaria*—a gram-negative plant bacterium—show promiscuous activity, converting d-galactose to d-KDGal through intermediates such as d-1,4-galactonolactone, facilitated by NAD(P)^+^-dependent l-arabinose 1-dehydrogenase (AraA) and l-arabinolactonase (AraB) (Peabody et al. 2019).

The subsequent steps in the bacterial pathway involve the phosphorylation of d-KDGal to d-KDGal 6-phosphate by d-KDGal kinase, leading to the production of d-glyceraldehyde 3-phosphate and pyruvate via d-KDGal 6-phosphate aldolase (Deacon and Cooper 1977) (Fig. 1). This phosphorolytic pathway mirrors the enzymatic reactions and products of d-KDGlc catabolism, demonstrating a conserved mechanism across bacterial species.

Furthermore, the first report of d-galactose catabolism through the DD pathway in the haloarchaeon *Haloferax volcanii* highlights the presence of this pathway not only in bacteria but also in specific archaeal species (Tästensen et al. 2020) (Fig. 1). This discovery underscores the broader application of the DD pathway, involving the phosphorylation and subsequent cleavage of d-KDGal into key metabolic intermediates across diverse microbes.

### Fungal d-galactonate pathway

In fungal metabolism, d-KDGal has been identified as a crucial metabolic intermediate within the d-galactonate pathway, as first reported by Elshafei and Abdel-Fatah (1991). Specifically, in *Aspergillus terreus*, the conversion of d-galactonate to d-KDGal is facilitated by d-galactonate dehydratase, which is a key step in this metabolic process (Fig. 1). Parallel to the catabolism of l-KDGal observed in the fungal pathway for d-galacturonate, the breakdown of d-KDGal by *A. terreus* involves a nonphosphorolytic reaction. This specific step is catalyzed by d-KDGal aldolase, which effectively splits d-KDGal into d-glyceraldehyde and pyruvate (Elshafei and Abdel-Fatah 1991) (Fig. 1).

## Phosphorolytic and nonphosphorolytic pathways for KDGal catabolism

The catabolism of KDGal in microorganisms occurs via two distinct pathways: phosphorolytic and nonphosphorolytic. These pathways represent different strategies for energy production and metabolic processing of KDGal, depending on the organism’s ecological niche and metabolic needs. The phosphorolytic pathway is utilized by certain species of bacteria and archaea, including *E. coli*, *A. vinelandii*, and *H. volcanii* (Deacon and Cooper 1977; Tästensen et al. 2020; Wong and Yao 1994). This pathway involves phosphorylation reactions that create high-energy phosphate bonds, such as KDGal 6-phosphate. These high-energy intermediates can drive subsequent metabolic reactions, leading to the efficient conversion of KDGal into common metabolic intermediates like pyruvate and glyceraldehyde 3-phosphate. The advantage of this pathway lies in its ability to produce energy more efficiently through these high-energy intermediates.

In contrast, the nonphosphorolytic pathway is observed in certain fungi and archaea, such as *T. reesei*, *A. niger*, *A. terreus*, and *Sulfolobus solfataricus*. In this pathway, KDGal is cleaved into glyceraldehyde and pyruvate. While pyruvate can enter central carbon metabolism for further catabolism, glyceraldehyde is reduced to glycerol, which may accumulate as a byproduct. This pathway may not be as energy efficient as the phosphorolytic pathway but offers other advantages.

On the other hand, the nonphosphorolytic pathway may confer greater metabolic flexibility, especially under conditions where phosphate availability is limited. Microorganisms employing this pathway can adapt to varying environmental conditions, which may provide a selective advantage in phosphate-scarce environments. Consequently, the choice of catabolic pathway might reflect the organism’s adaptation to its specific ecological niche and metabolic demands. In summary, the phosphorolytic and nonphosphorolytic pathways for KDGal catabolism highlight the metabolic diversity among microorganisms. The phosphorolytic pathway emphasizes energy efficiency through phosphorylation, while the nonphosphorolytic pathway offers adaptability in response to environmental constraints. Understanding these pathways enhances our knowledge of microbial metabolism and its ecological implications.

## Stereospecific methods for discrimination of enantiomers

### Polarimetry

Enantiomers are unique in that they share identical physical and chemical properties in a nonchiral environment, with the notable exception of their optical activity, that is, their ability to rotate the plane of polarized light in opposite directions (Díaz et al. 1998). Polarimetry, which exploits this singular distinction, is widely used to discern the enantiomeric nature of compounds (Kvittingen and Sjursnes 2020). However, the use of polarimetry to determine the enantiomeric composition of KDGal has not been explored.

A critical consideration in polarimetry is that only enantiomerically pure substances or enantiomeric excess mixtures can affect the plane of polarized light, whereas racemic mixtures or achiral substances do not exhibit optical activity. Consequently, the compound of interest must be isolated or enantiomerically enriched before optical rotation can reveal its enantiomeric nature (Cheng et al. 2022). Techniques, such as liquid chromatography coupled with polarimetric detection, have been used to achieve this separation (Lloyd and Goodall 1989).

One challenge inherent to polarimetry is the non-intuitive relationship between the direction of light rotation, denoted as positive ( +) or negative ( −), and the absolute configuration of the molecule (e.g., l- or d-configuration, *R*- or *S*-configuration). This discrepancy arises because molecules with identical stereochemical labels (l or d) can exhibit either positive or negative optical rotation. For instance, while d-sucrose displays a positive rotation, d-fructose shows a negative value despite both being d-enantiomers (Kvittingen and Sjursnes 2020). Accurate determination of the enantiomeric nature of a compound via polarimetry requires reference values for comparison or the use of purified enantiomers.

### Nuclear magnetic resonance spectroscopy

Nuclear magnetic resonance (NMR) spectroscopy is a pivotal analytical technique for assessing enantiomeric purity and determining the absolute configuration of compounds, and its utility has been well documented in various studies (Wenzel 2013). In exploring the chiral distinctions between the *erythro*- (d-KDGlc) and *threo*-forms (KDGal) of 2-keto-3-deoxy-hexonate, ^1^H-NMR spectroscopy has proven particularly insightful, enabling clear differentiation of these stereoisomers (Sakamoto et al. 2023; Yun et al. 2015).

Despite its powerful capabilities, NMR spectroscopy shares a common requirement with polarimetry—the necessity for analytes to be of high purity. This prerequisite often necessitates extensive preparatory work to isolate the target compound before analysis (Parker 1991). Moreover, the discrimination of enantiomeric structures via ^1^H-NMR typically involves chiral-specific derivatization. Chemical agents such as methoxy-α-trifluoromethyl-α-phenylacetic acid or α-methoxyphenylacetic acid are employed to facilitate this process, allowing for the enantiomers’ distinction (Seco et al. 2004).

The NMR spectroscopic landscape has evolved with the advent of innovative methodologies. Recent developments in parahydrogen-induced hyperpolarization have heralded a new era of NMR techniques, particularly for discriminating between the l- and d-forms of amino acids within complex mixtures (Dreisewerd et al. 2023). This advanced approach markedly reduces the need for the separation and purification steps traditionally required before ^1^H-NMR analysis, significantly streamlining the process (Dreisewerd et al. 2023).

### Stereospecific enzyme reaction

The intrinsic stereospecificity of enzyme–substrate interactions makes enzymatic methods particularly effective for discriminating between the enantiomeric forms of target compounds. Enzyme-based approaches have distinct advantages over other analytical methods. Unlike techniques that require the purification of compounds and specialized equipment such as polarimeters and NMR spectrometers, enzymatic reactions can directly discern enantiomers in reaction mixtures, significantly streamlining the process (Yu et al. 2022).

A critical factor in the success of enzymatic resolution is the inherent stereospecificity of an enzyme, which is determined by its structure and the functional groups within its active site. Therefore, selecting an enzyme that selectively reacts with one enantiomer over another (e.g., preferring the l-form to the d-form) is essential for accurate discrimination (Hilditch et al. 2007; Yu et al. 2022; Yun et al. 2023). Recent studies have highlighted the efficacy of this approach. In studies aimed at identifying the enantiomeric form of KDGal in the AHG catabolic pathway, l-KDGal aldolase (LGA1) from *T. reesei* has been used as a stereoselective enzyme. LGA1 exhibits activity exclusively toward l-KDGal, demonstrating that KDGal is present in this pathway as an l-enantiomer (Yun et al. 2023).

Furthermore, advances in enzyme engineering have shown that modifying specific amino acids in the active site of an enzyme can significantly enhance its stereospecificity (Royer et al. 2022). For instance, structural mutagenesis of the KDGlc aldolase from *S. solfataricus* resulted in a mutant variant (*Ss*KDG-aldolase variant 2) that altered the product ratio of d-KDGal to d-KDGlc from 45:55 in the wild type to 88:12. A different mutant variant (SsKDG-aldolase variant 4) demonstrated a preference for producing l-KDGal over l-KDGlc, changing the product ratio from 47:53 in the wild type to 76:24 (Royer et al. 2022).

## Perspectives for the production and utilization of l- and d-KDGal

### Production of l-KDGal as an inducer for pectin utilization

Recent studies have highlighted the pivotal role of l-KDGal as an inducer of the metabolic pathway for d-galacturonate utilization in *A. niger* (Alazi et al. 2017). Specifically, deletion of *gaaC*, which encodes l-KDGal aldolase in *A. niger*, leads to l-KDGal accumulation when d-galacturonate is provided as the carbon source. This genetic modification revealed that l-KDGal accumulates and acts as a regulatory molecule, significantly enhancing the expression of genes involved in d-galacturonate catabolism. These genes include *gaaA*, *gaaB*, and *gaaD*, which encode d-galacturonate reductase, l-galactonate dehydratase, and l-KDGal aldolase, respectively (Alazi et al. 2017).

Further transcriptome analysis of *A. niger ΔgaaC* grown on d-galacturonate demonstrated substantial upregulation of six pectinase genes. These enzymes play critical roles in breaking down pectin into oligomers or monomers, thereby facilitating the degradation of this complex polysaccharide. The significant expression of these pectinase genes in response to l-KDGal accumulation suggests a promising approach for enhancing the breakdown and utilization of pectin, a major component of waste biomass from fruit and vegetable processing (Alazi et al. 2017).

**Fig. 2:**
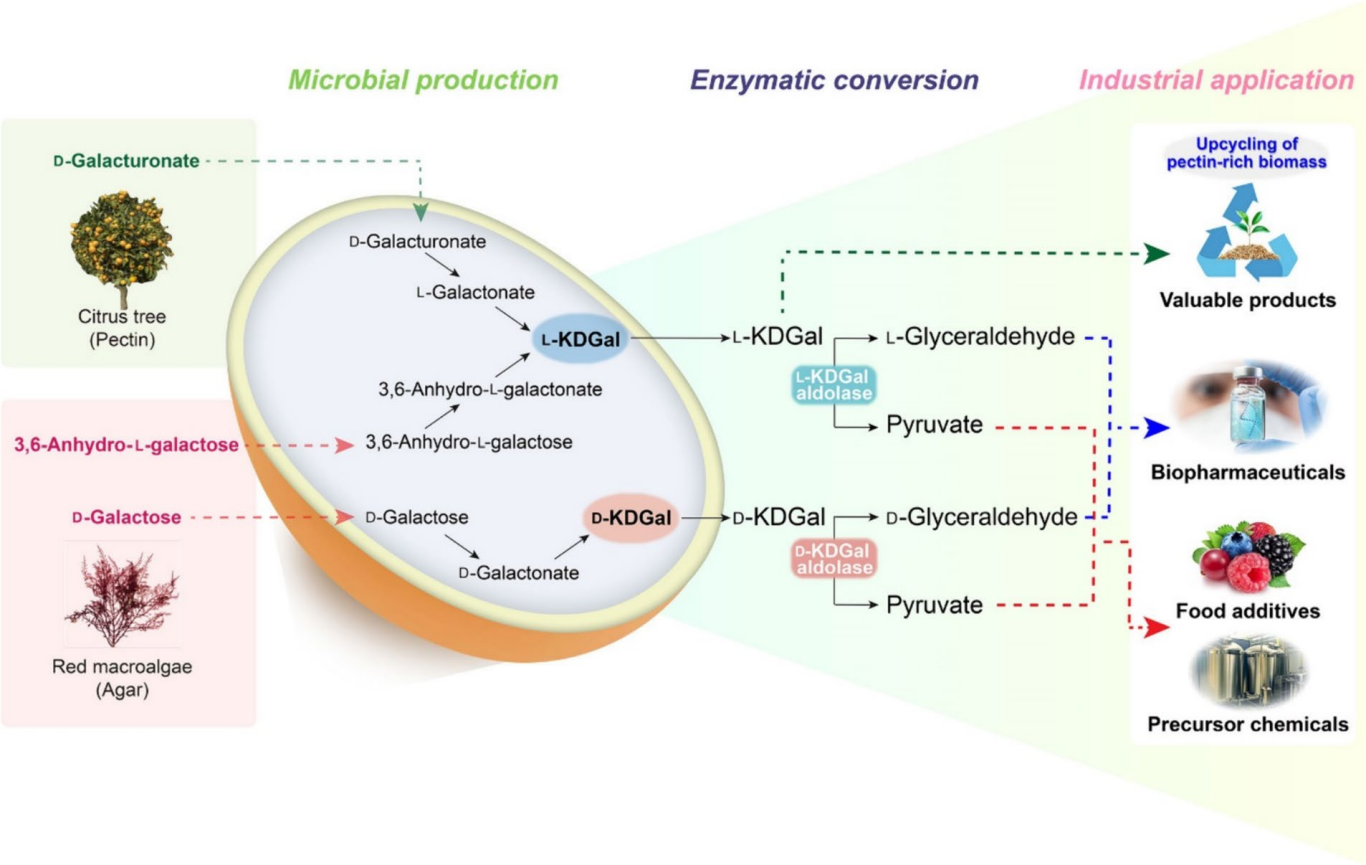
Strategies for producing KDGal and KDGal-derived products, pyruvate and glyceraldehyde, along with their industrial applications. Combining the microbial and enzymatic methods, in which the microbial production of l-KDGal or d-KDGal by engineered microbes followed by the one-step enzymatic conversion of l-KDGal or d-KDGal to pyruvate and l-glyceraldehyde or to pyruvate and d-glyceraldehyde using l-KDGal aldolase or d-KDGal aldolase, respectively, can be suggested as a promising strategy for the efficient production of KDGal and KDGal-derived products

Given the prevalence of pectin polysaccharides in agricultural and food waste, leveraging l-KDGal as an inducer of pectin degradation is an innovative and sustainable strategy. This approach not only enhances the efficiency of pectin-rich biomass utilization but also contributes to the generation of valuable materials from renewable resources, aligning with the current goals for waste valorization and the production of bio-based products (Roman-Benn et al. 2023) (Fig. 2).

### Pyruvate production

Pyruvate, a pivotal end product of glycolysis, has broad applications in the food, chemical, and pharmaceutical sectors because of its unique chemical properties and versatile functionality (Wada et al. 2020; Yuan et al. 2022) (Fig. 2). Recognized for its role in energy metabolism and as a potent antioxidant, pyruvate also serves as a critical precursor in the biosynthesis of bioplastics, isoprenes, alcohols, and amino acids (Yako et al. 2021; Wada et al. 2020).

The biological production of pyruvate has led to genetic engineering of various microbes, including *E. coli* (Moxley and Eiteman 2021), *Corynebacterium glutamicum* (Wieschalka et al. 2012), *Lactococcus lactis* (Suo et al. 2020), *Candida glabrata* (Luo et al. 2019), and *Klebsiella oxytoca* (Cao et al. 2020). Engineered strains of *L. lactis*, *C. glabrata*, and *K. oxytoca* have achieved pyruvate titers of 56.6 g/L, 53.1 g/L, and 71.1 g/L, respectively, using batch or fed-batch fermentation processes (Cao et al. 2020; Luo et al. 2019; Suo et al. 2020).

Pyruvate is a versatile metabolite with the capability to diverge into various metabolic pathways, including conversion into acetyl-CoA for the TCA cycle or conversion into acetaldehyde, lactate, and acetate. This versatility necessitates extensive genetic manipulation of microbial hosts to optimize pyruvate production, involving targeted modifications in pathways such as the TCA cycle, acetate, ethanol, amino acid synthesis, and lactate pathways (Luo et al. 2023; Moxley and Eiteman 2021; Suo et al. 2020; Wieschalka et al. 2012).

Moreover, the catabolic pathways of l-KDGal and d-KDGal offer an innovative one-step enzymatic approach for pyruvate production, utilizing KDGal aldolase to convert these substrates directly into pyruvate and glyceraldehyde without the need for cofactors (Fig. 2). A synergistic strategy that combines microbial and enzymatic methods, engineering microbes to produce l-KDGal or d-KDGal, followed by enzymatic conversion to pyruvate using specific aldolases, presents a promising avenue for enhancing pyruvate production efficiency (Fig. 2).

### l-Glyceraldehyde production

l-Glyceraldehyde, which can be synthesized from l-KDGal using l-KDGal aldolase, has emerged as a compound of interest in recent studies (Royer et al. 2022) (Fig. 2). A groundbreaking study revealed that l-glyceraldehyde can inhibit the growth of neuroblastoma cells and trigger apoptosis, marking a significant advancement in cancer research (Forbes et al. 2023). This inhibitory action extends to several cancer cell lines, including T98G brain cancer cells and human embryonic kidney (HEK)293 cells, as well as the neuroblastoma lines IMR-32 and SH-SY5Y, by impairing glycolysis and nucleotide metabolism (Forbes et al. 2023; Pietzke 2015).

l-Glyceraldehyde exhibits superior inhibitory effects on cancer cell growth compared with its d-isomer, highlighting its potential as a more effective agent in cancer therapeutics (Forbes et al. 2023). Moreover, the combination of 2-deoxy-d-glucose, a glucose analog known to hinder glucose metabolism in cancer cells, leads to energy depletion, and dl-glyceraldehyde has shown synergistic effects in curtailing tumor growth in Ehrlich ascites carcinoma models (Dey et al. 2022; Kapoor et al. 2014).

Thus, dl-glyceraldehyde is a promising bioactive material for developing biopharmaceuticals that inhibit cancer cell proliferation. This discovery not only underscores the therapeutic potential of l-glyceraldehyde but also opens new avenues for its application in cancer treatment strategies (Fig. 2).

### d-Glyceraldehyde production

d-Glyceraldehyde can be synthesized from d-KDGal using d-KDGal aldolase, a process detailed in seminal research by Theodossis et al. (2004) (Fig. 2). Early studies by Jain et al. (1975) revealed that d-glyceraldehyde possesses the unique ability to stimulate insulin secretion from isolated rat pancreatic islets. This effect was observed in both static incubation and perfusion systems, highlighting the potential of d-glyceraldehyde to modulate insulin levels (Jain et al. 1975).

In contrast, l-glyceraldehyde was found to lack the ability to initiate proinsulin biosynthesis, demonstrating significantly less efficacy in this regard than its d-enantiomer (Jain et al. 1975). These findings suggest distinct physiological roles and therapeutic potentials of the two enantiomers.

In light of these discoveries, both l- and d-glyceraldehyde have emerged as compounds of interest in pharmaceutical development and as bioactive materials. While l-glyceraldehyde shows promise in inhibiting tumor growth, d-glyceraldehyde offers potential benefits in inducing insulin production, presenting a novel therapeutic avenue for diabetes management (Fig. 2).

## Concluding remarks and future perspectives

KDGal, a common metabolic intermediate derived from the catabolism of pectin and red seaweed agar, demonstrates significant biochemical versatility and exists in both the l- and d-enantiomeric forms. The distinct catabolic pathways of these enantiomers, characterized by species-specific variations among bacteria, fungi, and archaea, utilize both nonphosphorolytic and phosphorolytic mechanisms. This nuanced understanding of KDGal catabolism has laid the groundwork for innovative biotechnological advancements, including developing engineered microbial platforms and enzymatic processes tailored for efficient substrate utilization.

Particularly, the role of l-KDGal in activating pectin catabolism genes positions it as a potent inducer of microbial production of high-value compounds from pectin-rich biomass. Furthermore, the conversion of KDGal to pyruvate, a compound of significant commercial interest across multiple industries, via a simple enantiomer-agnostic enzymatic step underscores the economic and industrial potential of KDGal as a precursor for bio-based chemical production.

Moreover, the bioactive derivatives of KDGal, namely, l- and d-glyceraldehydes, have emerged as compounds of therapeutic interest. l-Glyceraldehyde shows promise in oncology as a tumor growth inhibitor, whereas d-glyceraldehyde has potential applications in endocrinology, particularly in stimulating insulin production for diabetes management.

Efficiently producing KDGal presents a significant challenge in maintaining a balance of cofactors. The production of l-KDGal from d-galacturonate necessitates the presence of NAD(P)H. Conversely, the production of l-KDGal from AHG or d-KDGal from d-galactose requires NAD(P)^+^. Therefore, to sustain cofactor balance, it is crucial to explore and implement metabolic engineering strategies that integrate pathways consuming NAD(P)^+^ or NAD(P)H with those producing NAD(P)H or NAD(P)^+^, respectively. This integration will help maintain the necessary cofactor balance and enhance the efficiency of KDGal production.

In summary, KDGal is a valuable metabolic intermediate with broad application potential in producing value-added chemicals and therapeutic agents. As research continues to unravel the complexities of its catabolic pathways and explore innovative applications, KDGal remains at the forefront of biotechnological research and offers promising novel solutions to industrial, healthcare, and environmental challenges.

## Data Availability

All data generated or analyzed during this study are included in the submitted manuscript.
